# Direct production of polyhydroxybutyrate and alginate from crude glycerol by *Azotobacter vinelandii* using atmospheric nitrogen

**DOI:** 10.1038/s41598-022-11728-1

**Published:** 2022-06-07

**Authors:** Nobuhiro Yoshida, Ryuichi Takase, Yoshimi Sugahara, Yuko Nambu, Wataru Hashimoto

**Affiliations:** 1 Laboratory of Basic and Applied Molecular Biotechnology, Division of Food Science and Biotechnology, Graduate School of Agriculture, Kyoto University, Uji, Kyoto 611-0011 Japan; 2 South Incineration Plant, Kyoto City, Kyoto 612-8253 Japan

**Keywords:** Microbiology, Environmental sciences

## Abstract

While biodiesel is drawing attention as an eco-friendly fuel, the use of crude glycerol, a byproduct of the fuel production process, has increasingly become a concern to be addressed. Here we show the development of a low-cost fermentation technology using an atmospheric nitrogen-fixing bacterium to recycle crude glycerol into functional biopolymers. *Azotobacter vinelandii* showed substantial growth on tap water-diluted crude glycerol without any pretreatment. The number of viable *A. vinelandii* cells increased over 1000-fold under optimal growth conditions. Most of the glycerol content (~ 0.2%) in the crude glycerol medium was completely depleted within 48 h of culture. Useful polymers, such as polyhydroxybutyrate and alginate, were also produced. Polyhydroxybutyrate productivity was increased ten-fold by blocking the alginate synthesis pathway. Although there are few examples of using crude glycerol directly as a carbon source for microbial fermentation, there are no reports on the use of crude glycerol without the addition of a nitrogen source. This study demonstrated that it is possible to develop a technology to produce industrially useful polymers from crude glycerol through energy-saving and energy-efficient fermentation using the atmospheric nitrogen-fixing microorganism *A. vinelandii*.

## Introduction

Biodiesel is a fatty acid methyl ester obtained from the reaction of vegetable or animal oil/fat (triacylglycerol) with methanol in the presence of an alkaline catalyst^1^. In European countries, including France and Germany, the practical application of biodiesel is already underway as a national project, and it has become widespread in the society. Biodiesel is also actively produced in Japan. In particular, Kyoto, which has a large foodservice industry, is collecting waste cooking oil from restaurants and households. Biodiesel is also a low-pollution fuel, and compared with diesel oil, it significantly decreases the black smoke in automobile exhaust and produces almost no sulfur oxides that cause acid rain^1^. Thus, biodiesel is attracting attention as a carbon–neutral and eco-friendly fuel.

However, crude glycerol is generated as a byproduct of biodiesel production, which corresponds to approximately 10% of the material, and its use has become an issue. Crude glycerol contains many impurities and is alkaline in nature (pH 9.3). Because it is expensive to purify crude glycerol, there is an urgent need to find a way to use it^2^. Recently, many attempts have been made to recycle crude glycerol through microbial fermentation. Organic acids and mono- and diols are produced from crude glycerol by microbes^3–6^. However, impurities, including lipids and methanol, in crude glycerol and the high pH of crude glycerol interfere with the growth of many microorganisms. Therefore, pretreatments such as delipidation and neutralization are often necessary, and there are many problems in terms of cost for practical use^7^.

*Azotobacter vinelandii* can actively use atmospheric nitrogen for nitrogen fixation (N_2_ + 8H^+^  + 8e^−^  + 16 ATP → 2 NH_3_ + H_2_ + 16 ADP + 16 Pi) and needs no additional nitrogen source for growth. Therefore, it is expected to decrease the cost of cultivation during fermentation. *A. vinelandii* can use various substrates, including glycerol, as the sole carbon source. In addition, *A. vinelandii* shows substantial growth even at a higher pH^8,9^. Another remarkable property of *A. vinelandii* is its ability to synthesize biopolymers, such as polyhydroxybutyrate (PHB) and alginate. Many studies have reported on its industrial applications since its discovery approximately 100 years ago^10^.

PHBs are polyesters of 3-hydroxybutyric acid, which accumulate in cells as a carbon and energy source under starvation or oxygen limitation^11^. PHBs are expected to be used as materials for biodegradable plastics that microorganisms can degrade in the environment. The substitution of petroleum-based plastics with biodegradable plastics will solve the problems of waste and environmental pollution and decrease the large amount of CO_2_ released during the disposal process.

Alginate, an acidic polysaccharide composed of β-D-mannuronic acid (M) and α-L-guluronic acid (G), is produced by brown algae or certain bacteria (e.g., *Azotobacter* and *Pseudomonas*^12^). When water-soluble alginate forms salts with polyvalent cations such as Ca^2+^, the alginate solution loses its fluidity and is converted into a gel form. Alginate has many qualities, depending on the type of bound cation, M/G ratio, and degree of polymerization, and is used in various industries, for example as a food additive (thickening agent) and surgical thread^13^. Additionally, some M residues in bacterially derived alginate are acetylated, and alginate from *A. vinelandii* has a high frequency of G resides (G-block), which makes it gel easily^14^. Hence, alginate from *A. vinelandii* is more suitable for medical applications than that from brown algae^13^.

PHB and alginate have been previously produced from pure glycerol by *A. vinelandii* using atmospheric nitrogen^15^. This study used untreated crude glycerol as a sole carbon source for producing useful substances, such as PHB and alginate, by *A. vinelandii*.

## Results

### *A. vinelandii* assimilated crude glycerol diluted with water

**Figure 1:**
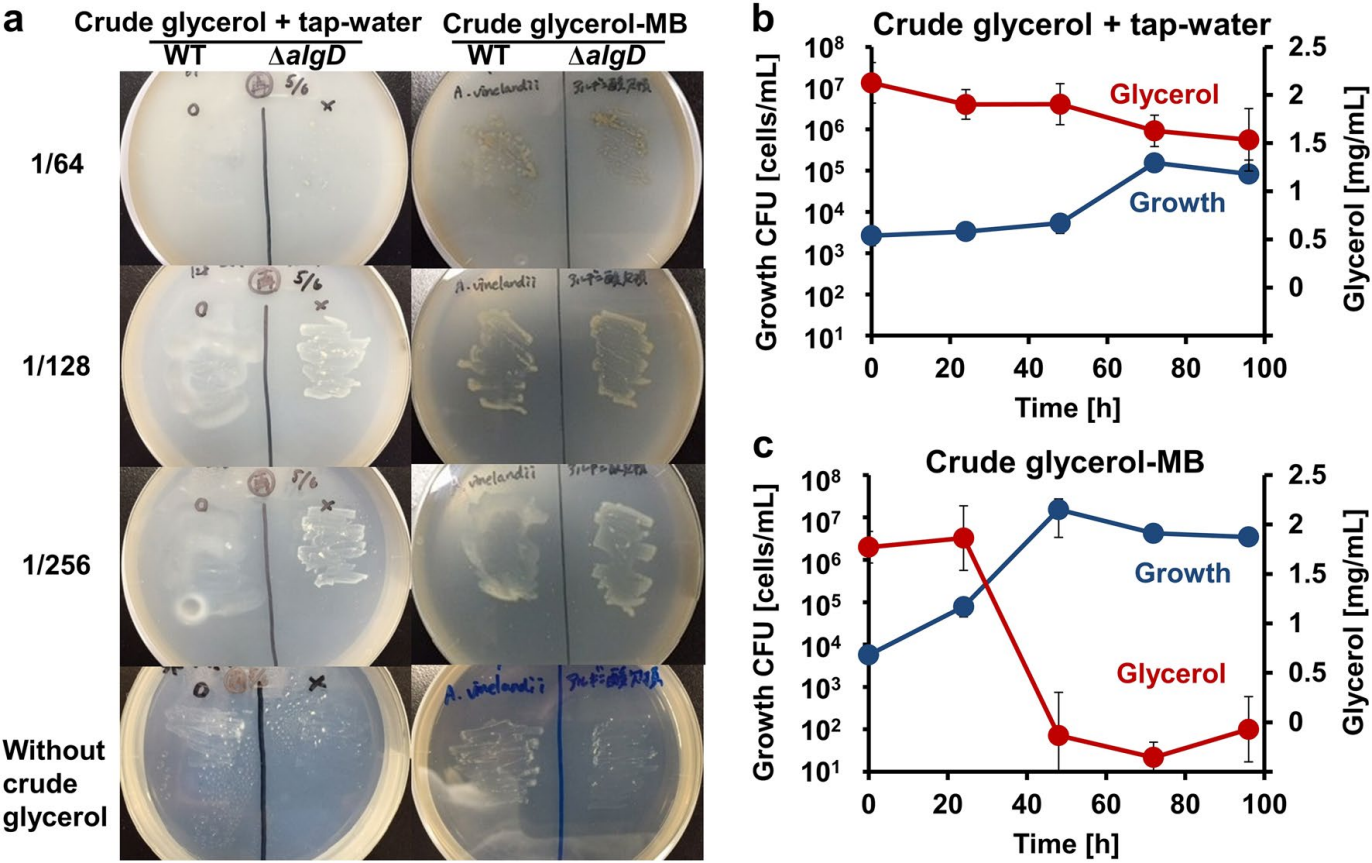
Crude glycerol assimilation by *A. vinelandii*. (**a**) Growth of *A. vinelandii* wild-type and Δ*algD* cells on crude glycerol plate medium. The labels (1/64, 1/128, and 1/256) on the left side represent the dilution fold. (**b**) Bacterial CFU (cells/mL) and residual glycerol concentration in 256-fold diluted crude glycerol medium. Blue and red-colored circles represent CFU and glycerol concentration, respectively. (**c**) Bacterial CFU (cells/mL) and residual glycerol concentration in 1/256-fold diluted crude glycerol-MB medium. Blue and red-colored circles represent CFU and glycerol concentration, respectively.

The growth of wild-type *A. vinelandii* was evaluated using crude glycerol medium plates with serial dilutions (16, 32, 64, 128, and 256-fold) of crude glycerol prepared using sterile tap water. Because *A. vinelandii* secretes viscous alginate, it may be challenging to count colonies during growth. Therefore, the growth of a strain lacking the alginate synthesis gene (*algD* encoding GDP-mannose 6-dehydrogenase) for comparison as a mutant Δ*algD* was also observed. As a result, there was little growth of wild-type and mutant Δ*algD* cells on the 16, 32, and 64-fold diluted medium plates, although wild-type and mutant Δ*algD* cells grew on the 128- and 256-fold diluted medium plates (Fig. 1a left). Both showed optimal growth on the 256-fold diluted medium plate. This result demonstrated that *A. vinelandii* can assimilate crude glycerol diluted with tap water, although impurities in crude glycerol above a certain concentration inhibited bacterial growth.

### Addition of minerals to the crude glycerol medium accelerated growth

In addition to the crude glycerol liquid medium diluted with tap water, multiple minerals commonly used in modified Burk’s (MB) medium for *A. vinelandii* were added. The growth of wild-type and mutant Δ*algD* cells was observed even at 64-fold dilution (Fig. 1a right). However, even in the absence of crude glycerol, a few bacterial colonies formed on the plate, suggesting that *A. vinelandii* can slightly assimilate agar as a sole carbon source. Subsequently, time-dependent growth was evaluated using a liquid medium. Simultaneously, residual glycerol concentration in the medium was also determined. Wild-type cells grew in 256-fold diluted crude glycerol with or without minerals for 96 h. Without adding minerals, cell numbers increased only by 100-fold at 96 h (Fig. 1b). Although the residual glycerol concentration reduced sequentially, some amount of glyercol was left in the culture. On the other hand, in the crude glycerol medium supplemented with minerals, including sodium, calcium, magnesium, iron, potassium, and phosphorus (crude glycerol-MB medium), cell numbers increased more than 1000-fold at 48 h. The glycerol concentration was depleted simultaneously (Fig. 1c). This result showed that adding minerals accelerated bacterial growth even in the liquid crude glycerol medium.

### Optimized growth conditions for crude glycerol assimilation

**Figure 2:**
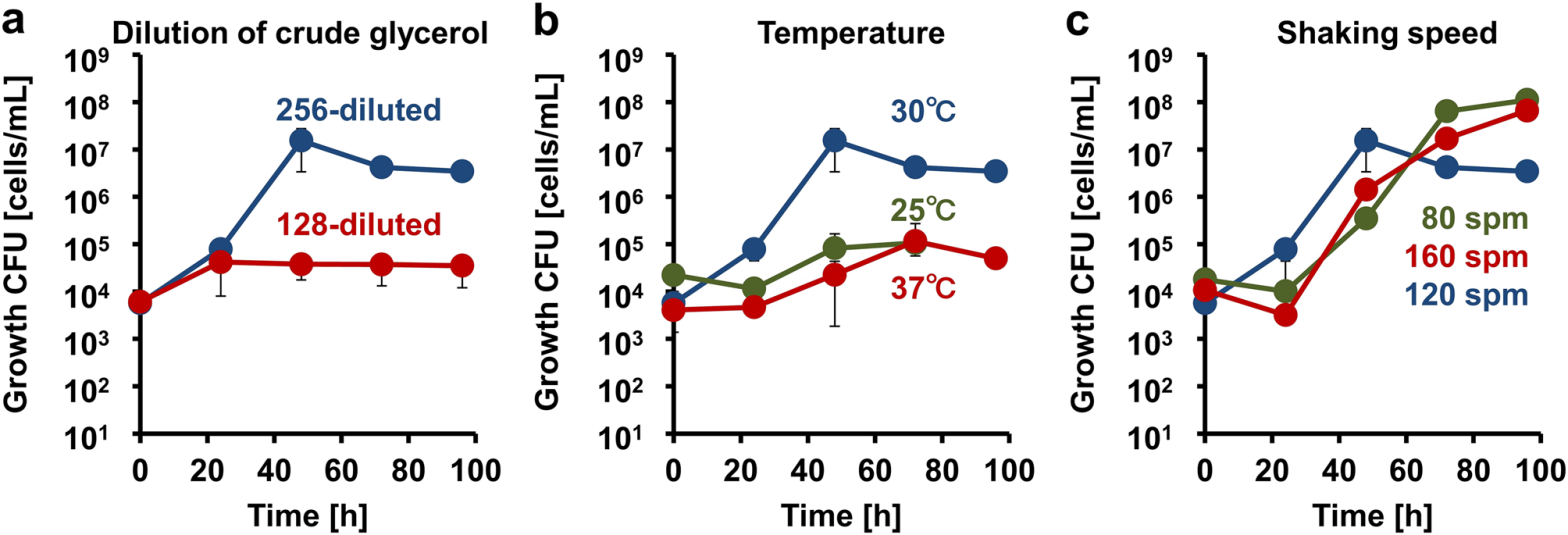
Assessment of culture conditions. (**a**) Effect of crude glycerol concentration on growth. Blue and red-colored circles represent 1/256 and 1/128-fold dilution, respectively. (**b**) Effect of temperature on growth. Blue, green, and red-colored circles represent 30, 25, and 37 °C, respectively. (**c**) Effect of shaking speed on growth. Blue, green, and red-colored circles represent 120, 80, and 160 spm, respectively.

To optimize the growth conditions, dilution, growth temperature, and shaking speed were varied in the liquid crude glycerol medium. Minerals were always added in the medium. To assess the effect of dilution, crude glycerol at 128-fold dilution was analyzed (Fig. 2a). However, growth was hardly detected. The optimum dilution factor was determined to be 256-fold because little growth was observed at concentrations beyond this owing to the lack of a carbon source. Subsequently, the incubation temperature was set at 25, 30, or 37 °C. Although growth was observed at every temperature, 30 °C was optimal (Fig. 2b). Finally, the optimal speed of the reciprocating shaker was evaluated at 80, 120, or 160 strokes per mim (spm), and it was observed that the speed had little effect on cell growth (Fig. 2c). From these results, the optimal growth conditions in crude glycerol-MB medium were 256-fold dilution, culture temperature of 30 °C, and shaking speed of 120 spm.

### Alginate production by *A. vinelandii* using crude glycerol

**Figure 3:**
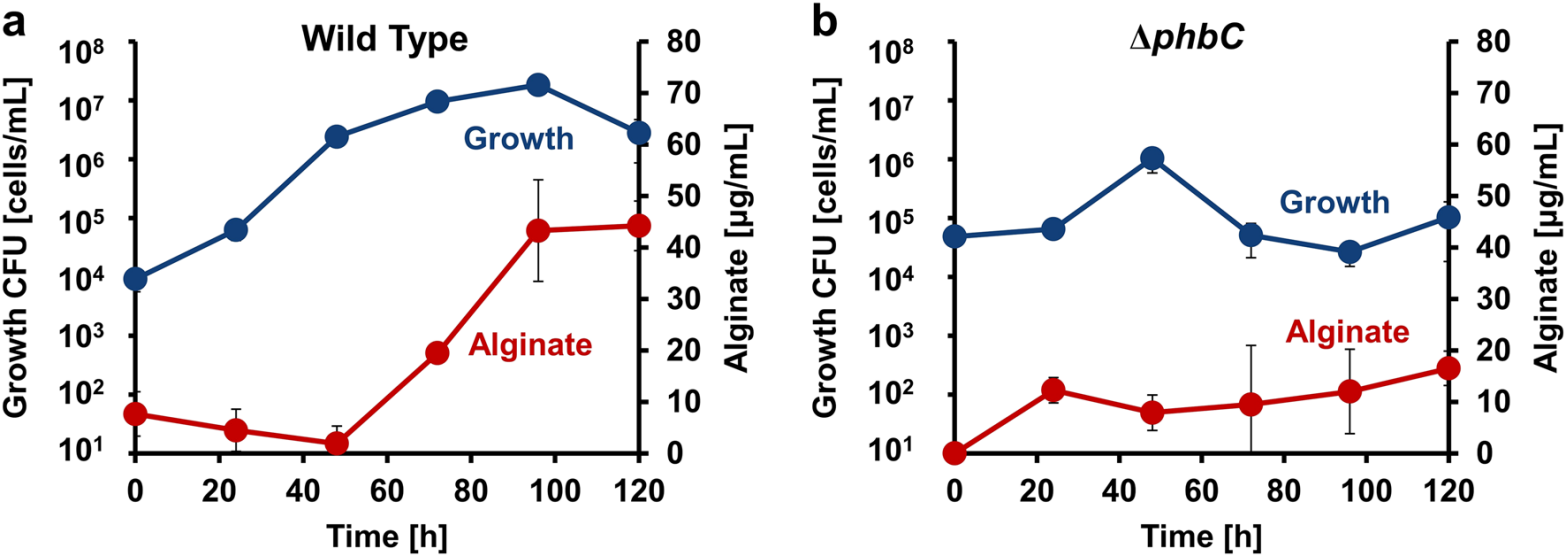
Alginate production from crude glycerol. (**a**) Growth and alginate production (wild-type cells). Blue and red-colored circles represent growth and alginate concentration, respectively. (**b**) Growth and alginate production (mutant Δ*phbC* cells). Blue and red-colored circles represent growth and alginate concentration, respectively.

Alginate production using crude glycerol was evaluated^16,17^. Because alginate production competes with PHB production, *phbC* crucial for PHB synthesis was disrupted. The resultant mutant Δ*phbC* cells as well as wild-type cells were subjected to alginate production assay. The wild-type cells began producing alginate after 48 h of culture, and the alginate concentration reached approximately 44 μg/mL at 120 h (Fig. 3a). The conversion rate from glycerol was 24 mg/g crude glycerol. This value is similar to that of alginate obtained from pure glycerol^15^. Alternatively, mutant Δ*phbC* cells hardly grew under the same conditions, and no alginate secretion was observed (Fig. 3b).

### PHB production from crude glycerol

**Figure 4:**
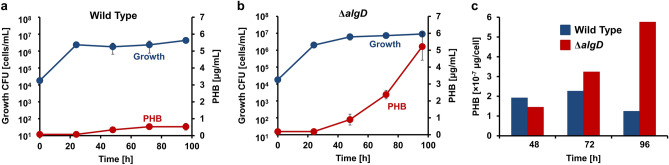
PHB production from crude glycerol. (**a**) Growth and PHB production (wild-type cells). Blue and red-colored circles represent growth and PHB concentration, respectively. (**b**) Growth and PHB production (mutant Δ*algD* cells). Blue and red-colored circles indicate growth and PHB concentration, respectively. (**c**) PHB production per cell. At each time point, blue and red-colored bars represent wild-type and mutant Δ*algD* cells, respectively.

*A. vinelandii* cells accumulate PHB as intracellular granules and use the polymer as a carbon source and energy source during starvation^10^. The amount of PHB was measured using gas chromatography. Because PHB production and alginate production compete with each other, mutant Δ*algD* cells in which the alginate synthesis pathway was disrupted were also evaluated. The wild-type and mutant Δ*algD* cells grown in crude glycerol-MB medium were harvested every 24 h for analyses. In both cells, PHB was detected after 48 h of culture (Fig. 4a,b). In the wild-type cells, the concentration of PHB was 0.53 μg/mL and cell numbers were 4.3 × 10^6^ cells/mL at 96 h. In mutant Δ*algD* cells, the concentration of PHB was 5.22 μg/mL and cell numbers were 9.0 × 10^6^ cells/mL at 96 h. The maximum PHB production per wild-type cell was 2.26 × 10^−7^ μg/cell at 72 h, which reduced after 96 h. Conversely, PHB production in mutant Δ*algD* cells increased up to 96 h, with a maximum value of 5.75 × 10^−7^ μg/cell (Fig. 4c). These results showed that blocking the alginate synthesis pathway is important to produce high amounts of PHB from crude glycerol.

### Morphological observation by electron microscopy

**Figure 5:**
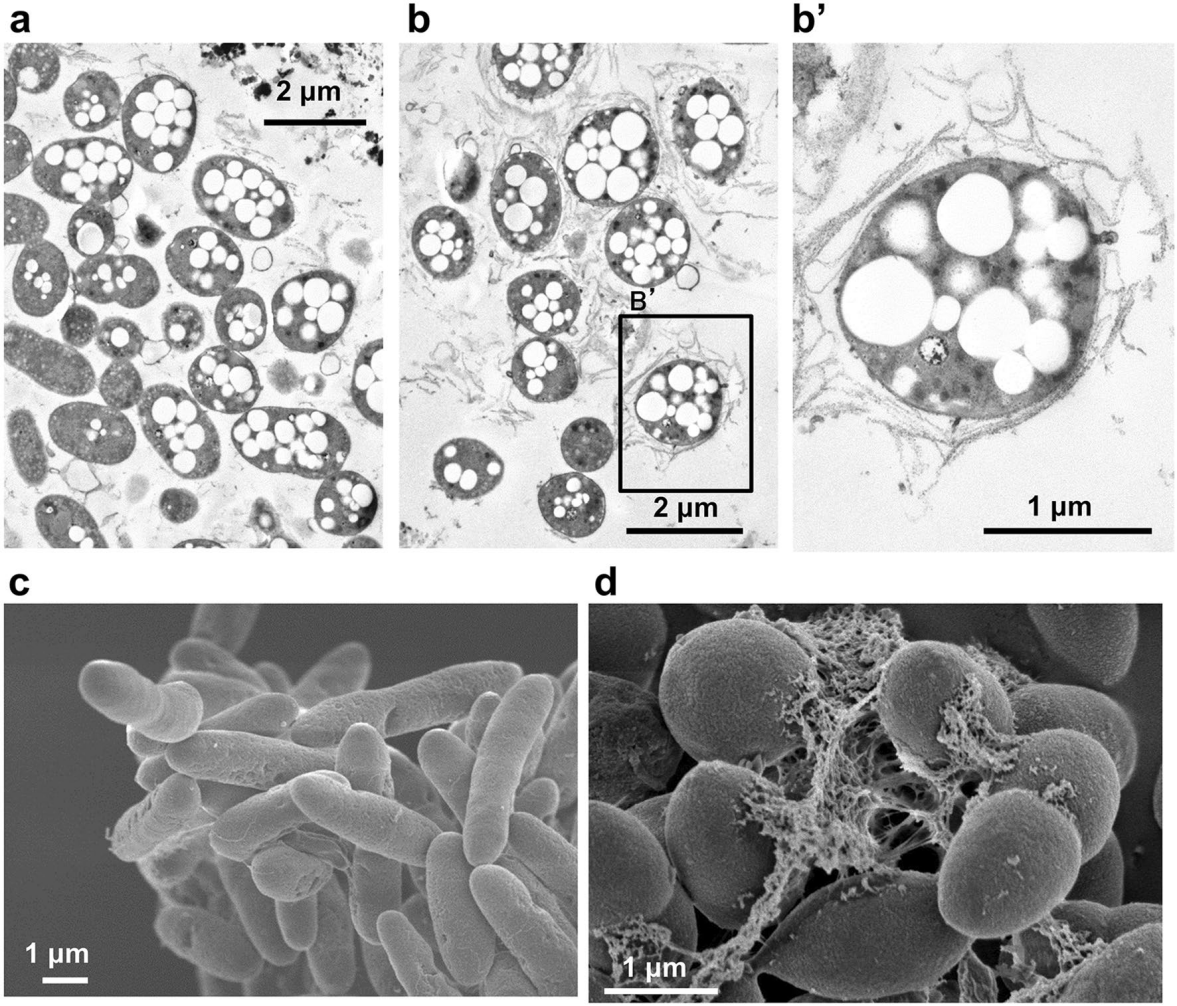
Electron microscopy images of *A. vinelandii* grown in crude glycerol medium. (**a**,**b**,**b’**) TEM images of cells cultured in crude glycerol-MB medium until the stationary growth phase (92 h). (**c**,**d**) SEM images of the surface of cells cultured in crude glycerol-MB medium [**c**: logarithmic growth phase (48 h), **d**: stationary phase (96 h)].

PHB is accumulated intracellularly as granules, whereas alginate is secreted extracellularly. These polymers are strongly related to cyst formation, which is induced by an unsuitable environment for growth. To observe whether cells grown in crude glycerol accumulate PHB, wild-type cells grown in the crude glycerol-MB medium were subjected to transmission electron microscopy (TEM). Most cells harvested at the stationary growth phase (92 h) accumulated abundant PHB granules inside the cells (Fig. 5a). In some cells, most of which were rod-shaped, no granules were observed (Fig. 5a). An extracellular membrane-like structure covering the cells was observed (Fig. 5b,b’). Scanning electron microscopy (SEM) revealed that cells in the logarithmic growth phase (48 h) were rod-shaped (Fig. 5c), whereas those in the stationary growth phase (96 h) were spherical with many fibrous materials between the cells (Fig. 5d).

## Discussion

*A. vinelandii* cells were demonstrated to assimilate crude glycerol diluted with tap water and produce useful substances, such as PHB and alginate. Their growth was improved by the addition of minerals, and PHB production was improved by blocking the alginate synthesis pathway. Although several studies have been reported on the production of alginate by *A. vinelandii* for industrial use, most of them used sugars, such as sucrose and glucose, as substrates^14^. Therefore, this study is the first to report on alginate production by *A. vinelandii* using “crude glycerol”, an industrial waste, as a substrate.

There are many PHB-producing bacteria (*Burkholderia cepacia*, *Cupriavidus necator*, *Pseudomonas putida*, *Bacillus megaterium*, etc.), and several reports on PHB production using “crude glycerol” as a substrate have been published^18–21^. To the best of our knowledge, the highest output of PHB per culture medium (59% cell dry weight) was reported by Freches et al. using a mixed microbial culture medium^22^. Although the production rate in this study is low compared with that reported by Freches et al., this is the first report on PHB production without using a nitrogen source. Because crude glycerol used in this study contained 45% glycerol, which is lower than that in commonly used (~ 70%)^6^, the production efficiency is expected to improve using crude glycerol with fewer impurities. The concentration of crude glycerol (256-fold dilution) that was determined to be optimal was approximately 0.2% in terms of glycerol concentration, which is low compared with the nutrient medium (~ 2%)^8^. Therefore, at present, alginate and PHB production is limited. It is important to identify the impurities that inhibit cell growth and devise a solution to this problem to enhance production efficiency. As shown in “Materials” section, 13% (w/w) methanol is included in the crude glycerol used in this study. Because methanol is known to inhibit various microorganisms^23^, removal of methanol seems to improve the growth of *A. vinelandii*. One possible future strategy to remove methanol from the crude glycerol is to co-culture methanotroph or methylobacterium that would remove the methanol.

Genetic manipulation is an effective approach to improve alginate production. In fact, the highest alginate production from *A. vinelandii* was reported by Mejia et al., who used a PHB synthesis-deficient mutant and cultured it under oxygen-controlled conditions^24^. Additionally, our laboratory has previously reported that a highly mucoid mutant obtained by introducing random mutations has about twice the alginate production capacity compared with the wild-type^15^. Also, in this study, a PHB synthesis-deficient mutant (Δ*phbC*) was created to increase alginate production. However, the cells grew poorly in the crude glycerol medium and exhibited little alginate secretion (Fig. 3b). This is because the cells were likely induced to become dormant cyst cells from the early stage of culture. It has been reported that blocking the PHB synthesis pathway results in cyst induction in *A. vinelandii*^25^. In this study, the colony-forming unit (CFU) measurements revealed that the growth of colonies of mutant Δ*phbC* cells was much slower than that of the colonies of wild-type cells. This suggests that a more rigorous examination of conditions is necessary when culturing PHB synthesis-deficient strains on crude glycerol medium.

PHB is actively produced in *A. vinelandii* cells during the logarithmic growth phase and is gradually consumed during the stationary growth phase or after cystification^26^. Wild-type and mutant Δ*algD* cells produce PHB after 48 h during the logarithmic growth phase (Fig. 4a,b). Compared with the wild-type cells, mutant Δ*algD* cells grew well and produced more PHB per cell (Fig. 4c). This may be because the carbon source used for alginate synthesis was restricted and used for growth or PHB synthesis.

## Conclusions and prospects

Recently, glycerol obtained as a byproduct of biodiesel production has been used to synthesize a value-added product, triacetin, which is a fuel additive. In certain studies, glycerol was esterified with acetic acid to convert it into triacetin through catalytic conversion^27,28^. There are several ways to enhance the economic viability of biodiesel. Not only biopolymers but also other useful substances can be produced from crude glycerol using microorganisms. Furthermore, the assessment of sustainability is an emerging research area of interest. The developed techniques, such as those used for producing useful substances, should be compatible with the environment. Till date, several advanced tools to assess sustainability based on the concepts of emergy, life cycle assessment, energy, and exergy have been developed^29,30^. Hence, we should aim to improve biopolymer production from crude glycerol in a more sustainable manner.

**Figure 6:**
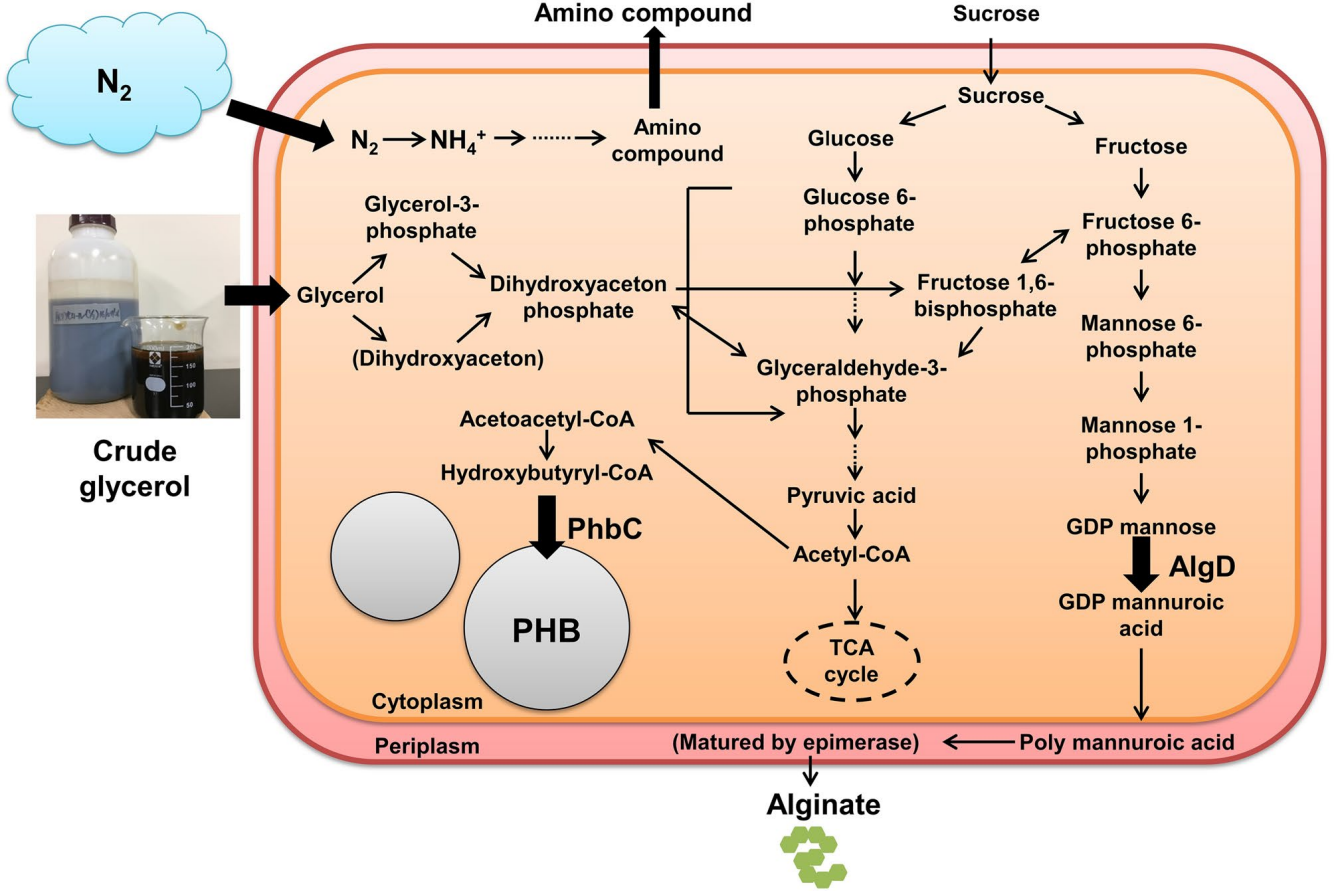
Model and metabolic map of crude glycerol recycling in *A. vinelandii*.

In conclusion, our study is the first step in the development of a technology for recycling crude glycerol through energy-saving and energy-efficient fermentation using the atmospheric nitrogen-fixing microorganism *A. vinelandii* (Fig. 6).

## Methods

### Materials

Crude glycerol used in this study was provided by the Kyoto Municipal Waste Edible Oil Fuel Production Facility (https://www.city.kyoto.lg.jp/kankyo/page/0000065549.html). It comprised 45% (w/w) glycerol, 13% (w/w) methanol, 25% (w/w) lipids, and other impurities such as 2.7% (w/w) potassium, 0.92% (w/w) water, 0.02% (w/w) nitrogen, and 0.01% (w/w) sulfur. Sodium alginate (average molecular weight: 300,000) from *Eisenia bicyclis* and agar were purchased from Nacalai Tesque (Kyoto, Japan). Restriction endonucleases and DNA-modifying enzymes were obtained from Toyobo (Osaka, Japan) or Takara Bio (Shiga, Japan). All other analytical-grade chemicals used herein were commercially available.

### Bacterial strains and culture

In this study, the following bacterial strains were used: *A. vinelandii* ATCC12837 wild-type, an alginate synthesis-deficient mutant (Δ*algD*) with disrupted *algD* encoding GDP-mannose 6-dehydrogenase^15^, and a PHB synthesis-deficient strain (Δ*phbC*) with disrupted *phbC* encoding polyhydroxyalkanoate synthase subunit. The bacterial cells were grown aerobically in crude glycerol-MB medium (200 µg/mL NaCl, 50 µg/mL CaSO_4_, 200 µg/mL MgSO_4_·7H_2_O, 2.9 µg/mL Na_2_MoO_4_·2H_2_O, 27 µg/mL FeSO_4_·7H_2_O, 0.66 mg/mL K_2_HPO_4_, and 0.16 mg/mL KH_2_PO_4_) containing crude glycerol as a sole carbon source. For the medium plate, agar was added at a concentration of 1.5% (w/v).

### Disruption of *phbC* in *A. vinelandii*

To amplify *phbC* (1.7 kb) in the genomic DNA of *A. vinelandii* cells, polymerase chain reaction (PCR) was performed using KOD-Plus-Neo polymerase (Toyobo) and two synthetic oligonucleotide primers containing an NdeI (forward) or XhoI site (reverse) at their 5′ ends (forward, 5′-GGCATATGGATCAAGCCCCCTCTTTCACAAGTTTC-3′; reverse, 5′-GGCTCGAGGCCTTTCACGTAACGGCCTGGTGCTGCCTC-3′) (Hokkaido System Science, Sapporo, Japan). The PCR product was cloned into HincII-digested pUC119 (Takara Bio) using Ligation High (Toyobo). The tetracycline-resistance gene (*tet*^r^, 1.9 kb) was amplified from plasmid pACYC184 (Nippon Gene, Tokyo, Japan) using two synthetic oligonucleotide primers (forward, 5′-GGATTCTCATGTTTGACAGCTTATCATCGA-3′; reverse, 5′-CCCTACCGGACAGCGGTGCGGACTGTTGTA-3′). The amplified *tet*^r^ fragment was inserted into the middle of *phbC* in pUC119-*phbC* at the HincII restriction site using Ligation High (Toyobo). The resultant *tet*^r^-inserted *phbC* (*phbC*::*tet*^r^) fragment was amplified using two synthetic oligonucleotide primers used for *phbC* amplification.

*A. vinelandii* cells were transformed via natural transformation. The cells were grown in MB medium (containing 1% glucose instead of crude glycerol as a carbon source) at 30 °C until the exponential growth phase. Subsequently, 50 µL of the culture was mixed with 5 µL of *phbC*::*tet*^r^ fragment solution (1.0 µg/µL). This solution was inoculated on a 0.22-µm membrane filter placed on MB medium plate containing 1% glucose. After 24 h of incubation at 30 °C, the cells on the filter were suspended in 1 mL of MB buffer (50 µg/mL CaSO_4_, 200 µg/mL MgSO_4_·7H_2_O, 2.9 µg/mL Na_2_MoO_4_·2H_2_O, 27 µg/mL FeSO_4_·7H_2_O, 0.66 mg/mL K_2_HPO_4_, and 0.16 mg/mL KH_2_PO_4_). The suspension was inoculated onto MB medium plate containing 2% sucrose and 10 µg/mL tetracycline hydrochloride. After 3 days of incubation at 30 °C, tetracycline-resistant colonies were isolated. The disrupted *phbC* sequence was confirmed through amplification using PCR and dideoxy chain termination using an automated DNA sequencer (3130Xl Genetic Analyzer, Thermo Fisher Scientific, Waltham, MA, USA).

### Glycerol assay

Glycerol concentration in the medium was measured via an enzyme-based method using F-kit glycerol (J.K. International, Tokyo, Japan), as described previously^31^.

### Alginate assay

The amount of alginate in the culture broth was measured using the method described by Sabra^16^. Briefly, 300 µL of the culture broth was mixed with 12 µL of 0.5 M EDTA and 6 µL of 5 M NaCl. Subsequently, alginate was separated from the cells using centrifugation (17,000 × *g*, 4 °C, 5 min). Then, 280 µL of the supernatant was mixed with 900 µL of cold isopropanol, followed by incubation on ice for 10 min. After centrifugation (17,000 × *g*, 4 °C, 5 min), the pellet was washed with cold 70% ethanol and dissolved in 1 mL of sterile water at 4 °C. The resultant solution was used as the sample for alginate assay. The amount of alginate was measured according to the carbazole sulfate method^17^. The sample was mixed with a cold solution comprising 735 µL of sulfuric acid, 17.5 µL of boric acid solution (45 mM KOH and 1 M boric acid), and 25 µL of 0.1% (w/v) carbazole. The mixture was incubated at 55 °C for 30 min, following which its absorbance was measured at 530 nm. The standard curve was obtained using sodium alginate.

### PHB assay

Briefly, 30 mL of the culture was centrifuged (4300 × *g*, 5 min), and the obtained pellet was resuspended in 1 mL of sterile water. The suspension was freeze–dried. Subsequently, 1 mL of chloroform containing 0.5% (w/v) benzoic acid and 1 mL of methanol containing 3% sulfuric acid was added to the dried cells. The solution was heated at 100 °C for 140 min. After adding 1 mL of sterile water, the solution was vortexed for 1 min and centrifuged (3000 × *g*, 4 °C, 5 min). The lower organic layer was subjected to gas chromatography on GC-2014 (Shimadzu, Kyoto, Japan) using a DB-5 column (30 m × 0.25 mm × 0.25 μm, Agilent Technologies, Santa Clara, CA, USA) and helium as the carrier gas. The temperatures of the detector and injector were set at 275 and 230 °C, respectively. The oven temperature was raised from 60 to 230 °C at a rate of 8 °C/min during analysis. (*R*)-3-Hydroxybutyrate (Sigma, St. Louis, MO) was used as a standard.

### TEM

TEM was conducted at Tokai Electron Microscopy Analysis Co. (Nagoya, Japan). Briefly, 30 mL of *A. vinelandii* culture grown in crude glycerol-MB medium for 92 h was centrifuged (4300 × *g*, 5 min) and the pellet was resuspended in 5 mL of saline. For prefixation, the suspension was mixed with an equal volume of fixation solution A [2% paraformaldehyde, 2% glutaraldehyde, and 0.1 M potassium phosphate buffer (pH 7.4)], followed by incubation at 4 °C for 60 min. Subsequently, the suspension was centrifuged (4300 × *g*, 4 °C, 5 min) and the pellet was suspended in fixation solution B [2% osmium tetroxide and 0.1 M potassium phosphate buffer (pH 7.4)], followed by incubation at 4 °C for 24 h. The cells were centrifuged to remove the fixation solution, washed with 2-[4-(2-hydroxyethyl)-1-piperazinyl]ethanesulfonic acid (HEPES) buffer, and treated with HEPES buffer containing 2% osmium tetroxide postfixation. The cells were then dehydrated in a step-wise manner using 30, 50, 70, 90, and 100% ethanol. The solvent was displaced by treatment with propylene oxide, and the Quetol-812 resin (Nisshin EM, Tokyo, Japan) was added for polymerization at 60 °C for 2 days. Then, ultrathin sections of 65–70-nm thickness were prepared from cells using a 2088 ULTROTOME V (LBK) diamond knife. Finally, the cells were stained using 2% uranyl acetate and lead, and the cell section structures were observed via TEM (JEM-1200EX; 80 kV, JEOL, Tokyo, Japan).

### SEM

Briefly, 5 mL of the culture medium was mixed with an equal volume of 4% paraformaldehyde, followed by incubation for 2 h at 4 °C for prefixation. The mixture was centrifuged (17,000 × *g*, 4 °C, 5 min), and the pellet was washed thrice with sterile water (4 °C, 30 min). Subsequently, 1 mL of 1% osmium oxide was added and mixed, followed by incubation for 2 h at 4 °C postfixation. The solution was subjected to centrifugation (17,000 × *g*, 4 °C, 5 min), the supernatant was removed, and the pellet was washed with sterile water at 4 °C for 2 h. Then, dehydration was performed in a step-wise manner using 50, 70, 90, and 100% ethanol. After centrifugation, the pellet was treated twice with *t*-butyl alcohol for 30 min at room temperature and was centrifuged again. Following this, the pellet was suspended in a minimal amount of *t*-butyl alcohol and frozen at 4 °C. The frozen samples were subjected to decreased-pressure drying and completely dried. The obtained samples were coated with platinum–palladium and observed via SEM (Hitachi SU8230, Hitachi, Tokyo, Japan).
